# Supplementary material for: Bacterial DNA on the skin surface overrepresents the viable skin microbiome
Source: eLife. 2023 Jun 30;12:RP87192. doi: 10.7554/eLife.87192 (PMC10328497; doi:10.7554/eLife.87192)
Supplement: Supplementary file 1. [file elife-87192-supp1.docx]

| **Name** | **Description** | **Sequence (5' - 3')** | **Reference** |
| --- | --- | --- | --- |
|  | ddPCR FP (Universal bacterial 16S qPCR FP) | TCCTACGGGAGGCAGCAGT | (*37*) |
|  | ddPCR RP (Universal bacterial 16S qPCR RP) | GGACTACCAGGGTATCTAATCCTGTT | (*37*) |
| EFTU_FP | Staph-specific ddPCR forward primer | ATGCCACAAACTCGTGAACA | this paper |
| EFTU_RP | Staph-specific ddPCR reverse primer | ACATCGTCACCTGGGAAGTC | this paper |
| EUB338 | Pan-bacterial FISH probe | GCTGCCTCCCGTAGGAGT | (*17*) |
| NonEUB338 | Nonsense control FISH probe | CGACGGAGGGCATCCTCA | (*38*) |
|  | C. acnes FISH probe | GAGTGTGTGAACCGATCATGTAGTAGGCAA | (*39*) |
| 27F | Forward 16S sequencing primer | AGAGTTTGATCCTGGCTCAG | (*40*) |
| 534R | Reverse 16S sequencing primer | ATTACCGCGGCTGCTGG | (*40*) |

**Supplementary file 1.** Nucleotide sequences used in this study.
