# Supplementary material for: Bacterial DNA on the skin surface overrepresents the viable skin microbiome
Source: eLife. 2023 Jun 30;12:RP87192. doi: 10.7554/eLife.87192 (PMC10328497; doi:10.7554/eLife.87192)
Supplement: Supplementary file 2. [file elife-87192-supp2.docx]

| **Sample** | **Sequence count** |
| --- | --- |
| **HV4 Nares +PMA** | **153847** |
| **HV1 Nares +PMA** | **114649** |
| **HV1 RAC +PMA rep1** | **108308** |
| **HV2 Glabella +PMA** | **101272** |
| **Hair shaft +PMA rep2** | **97416** |
| **HV3 Glabella -PMA** | **76165** |
| **HV1 Glabella -PMA rep4** | **70341** |
| **HV2 Nares -PMA** | **69549** |
| **HV2 Glabella -PMA** | **67565** |
| **HV1 Glabella -PMA rep1** | **61693** |
| **HV1 TW -PMA** | **61628** |
| **HV3 Glabella -PMA** | **60889** |
| **HV3 RAC -PMA** | **60330** |
| **HV2 AF -PMA** | **59564** |
| **HV1 RAC -PMA rep2** | **59158** |
| **HV2 PF -PMA** | **58842** |
| **HV1 Glabella -PMA rep3** | **56854** |
| **HV4 RAC -PMA** | **54039** |
| **HV2 Back -PMA** | **53930** |
| **HV3 Nares +PMA** | **53143** |
| **HV2 VF -PMA** | **52215** |
| **Hair shaft -PMA** | **51698** |
| **HV3 RAC -PMA** | **50870** |
| **HV4 Glabella -PMA** | **49799** |
| **HV4 RAC +PMA** | **49501** |
| **HV2 RAC -PMA** | **49497** |
| **HV3 VF -PMA** | **48957** |
| **HV3 Nares -PMA** | **48546** |
| **HV3 Nares +PMA** | **48511** |
| **HV4 Nares -PMA** | **47917** |
| **HV3 Back -PMA** | **46631** |
| **HV1 Nares -PMA** | **46250** |
| **HV3 RAC +PMA** | **45487** |
| **HV3 AF -PMA** | **43929** |
| **HV2 Nares +PMA** | **43324** |
| **HV1 RAC -PMA rep1** | **42463** |
| **HV2 RAC +PMA** | **40333** |
| **HV1 Back -PMA** | **39487** |
| **HV3 nares -PMA** | **39419** |
| **HV1 Glabella +PMA rep4** | **39357** |
| **HV1 Glabella +PMA rep2** | **38231** |
| **HV1 TW +PMA** | **36139** |
| **HV1 Glabella -PMA rep2** | **35249** |
| **HV3 RAC +PMA** | **34519** |
| **HV1 Glabella +PMA rep3** | **34273** |
| **HV1 Back +PMA** | **34201** |
| **HV3 Glabella +PMA** | **32629** |
| **HV3 PF -PMA** | **31665** |
| **HV1 Glabella +PMA rep1** | **26953** |
| **HV1 RAC +PMA rep2** | **25388** |
| **HV1 PF -PMA** | **20970** |
| **Hair shaft -PMA rep2** | **18791** |
| **HV4 Glabella +PMA** | **18636** |
| **HV1 PF +PMA** | **17414** |
| **Hair shaft -PMA rep1** | **17389** |
| **HV3 Back -PMA** | **17115** |
| **HV3 AF -PMA** | **16969** |
| **HV4 VF -PMA** | **16956** |
| **HV4 PF -PMA** | **15653** |
| **HV1 VF -PMA** | **15134** |
| **HV3 AF +PMA** | **14639** |
| **Hair shaft -PMA rep1** | **14192** |
| **HV3 VF +PMA** | **9156** |
| **Hair shaft +PMA** | **9136** |
| **HV1 AF -PMA** | **9096** |
| **HV4 PF +PMA** | **7483** |
| **HV3 PF +PMA** | **7438** |
| **HV2 Back +PMA** | **6296** |
| **HV4 AF -PMA** | **6261** |
| **HV3 PF -PMA** | **6154** |
| **HV2 VF +PMA** | **6032** |
| **Hair shaft +PMA rep1** | **5321** |
| **HV3 VF -PMA** | **5281** |
| **HV2 PF +PMA** | **5095** |
| **HV4 Back -PMA** | **4793** |
| **blank rep1** | **4781** |
| **HV3 Glabella +PMA** | **4593** |
| **Hair shaft +PMA rep1** | **4428** |
| **HV3 Back +PMA** | **4286** |
| **HV3 Back +PMA** | **4191** |
| **HV3 VF +PMA** | **4180** |
| **HV1 VF +PMA** | **3596** |
| **HV4 Back +PMA** | **3315** |
| **blank rep2** | **3291** |
| **Hair shaft -PMA rep2** | **3053** |
| **HV3 AF +PMA** | **2647** |
| **Hair shaft +PMA rep2** | **1819** |
| **HV4 VF +PMA** | **1408** |
| **HV3 PF +PMA** | **1111** |
| **HV2 AF +PMA** | **1033** |
| **HV4 AF +PMA** | **572** |
| **HV1 AF +PMA** | **421** |

**Supplementary file 2.** Sequence counts for Figures 2-4.
