# Supplementary material for: Bacterial DNA on the skin surface overrepresents the viable skin microbiome
Source: eLife. 2023 Jun 30;12:RP87192. doi: 10.7554/eLife.87192 (PMC10328497; doi:10.7554/eLife.87192)
Supplement: Supplementary file 3. [file elife-87192-supp3.docx]

| **C. acnes** | **527328** |
| --- | --- |
| **Skin cocktail** | **500040** |
| **V2.3 -PMA** | **492270** |
| **V1.2 -PMA** | **455368** |
| **V3.0 -PMA** | **450136** |
| **V3.0 +PMA** | **446767** |
| **V1.1 -PMA** | **437976** |
| **V3.1 -PMA** | **434868** |
| **V4.3 -PMA** | **412972** |
| **V2.0 -PMA** | **403077** |
| **V3.2 -PMA** | **379443** |
| **V5.0 -PMA** | **376615** |
| **C. striatum** | **375535** |
| **V3.3 -PMA** | **366751** |
| **V2.2 -PMA** | **363327** |
| **V5.3 -PMA** | **338217** |
| **V2.1 -PMA** | **334685** |
| **S. epidermidis** | **328603** |
| **V1.1 +PMA** | **315313** |
| **V5.2 -PMA** | **313142** |
| **V1.2 +PMA** | **312954** |
| **V1.3 -PMA** | **311212** |
| **V1.3 +PMA** | **309985** |
| **V4.2 -PMA** | **307476** |
| **V4.0 -PMA** | **306340** |
| **V2.1 +PMA** | **298029** |
| **V1.0 +PMA** | **288799** |
| **V2.3 +PMA** | **285720** |
| **V4.2 +PMA** | **280293** |
| **V4.1 -PMA** | **255623** |
| **V3.1 +PMA** | **254358** |
| **V4.3 +PMA** | **248363** |
| **V1.0 -PMA** | **244772** |
| **M. luteus** | **233452** |
| **PBS 0 +PMA** | **227814** |
| **V5.0 +PMA** | **218913** |
| **V5.1 -PMA** | **217713** |
| **V2.0 +PMA** | **211122** |
| **V4.1 +PMA** | **200852** |
| **V5.2 +PMA** | **198699** |
| **V4.0 +PMA** | **197961** |
| **V3.2 +PMA** | **197419** |
| **PBS 3 -PMA** | **183592** |
| **V3.3 +PMA** | **175750** |
| **V2.2 +PMA** | **157845** |
| **PBS 2 -PMA** | **133797** |
| **PBS 0 -PMA** | **117178** |
| **PBS 3 +PMA** | **106895** |
| **PBS 1 +PMA** | **91483** |
| **V5.3 +PMA** | **51933** |
| **PBS 2 +PMA** | **35707** |
| **V5.1 +PMA** | **32808** |
| **PBS 1 -PMA** | **25336** |

**Supplementary file 3**. Sequence counts in perturbation recovery
